# Real world practice indirect comparison between guselkumab and risankizumab: Results from an Italian retrospective study

**DOI:** 10.1111/dth.15214

**Published:** 2021-11-30

**Authors:** Angelo Ruggiero, Gabriella Fabbrocini, Eleonora Cinelli, Matteo Megna

**Affiliations:** ^1^ Section of Dermatology, Department of Clinical Medicine and Surgery University of Naples Federico II Naples Italy

**Keywords:** anti‐IL‐23, biologics, guselkumab, indirect comparison, psoriasis, real world, risankizumab

## Abstract

IL‐23‐inhibitors, such as guselkumab and risankizumab, represent the newest class of biologics approved for psoriasis. Phase III trials have shown their efficacy and safety. However, real life data are still scant. to indirectly compare the effectiveness, safety and tolerability of guselkumab and risankizumab in real world practice. An Italian single‐center retrospective cohort study enrolling moderate‐to‐severe psoriasis patients from September 1, 2018 and December 31, 2020 was performed to indirectly compare guselkumab and risankizumab efficacy and safety. Sixty eight patients were included (36 received guselkumab and 32 risankizumab). The groups were comparable for all analyzed characteristics, except for mean psoriasis duration (*p* < 0.01) which was higher for guselkumab. In guselkumab group, mean PASI reduced from 16.1 ± 6.4 (baseline) 2.1 ± 0.9 (week‐28) (*p* < 0.001) up to 0.9 ± 0.8 (week‐44) (*p* < 0.001). In risankizumab group mean PASI decreased from 13.5 ± 4.9 (baseline) 1.9 ± 0.8 (*p* < 0.001), (week‐28) (*p* < 0.001) up to 0.9 ± 0.4 (week‐40) (*p* < 0.001). No significant difference in mean PASI and BSA were observed between the treatments. No cases of serious AEs, injection site reaction, candida, malignancy, cardiovascular events were reported in both groups. Guselkumab and risankizumab showed favorable efficacy and safety profile, being comparable in terms of PASI90 and PASI100 responses as well as in AEs frequency and discontinuation rates.

## 1 INTRODUCTION

Psoriasis is a chronic inflammatory skin disease that may be associated with numerous comorbidities, resulting in a considerable impact on patients' quality of life.^1^ Psoriasis pathogenesis is complex with a unique trigger or aetiologic factor being not detected. Recent major research advantages lead to the development of biologic therapies targeting specific cytokines engaged in the chronic inflammation which sustain psoriasis.^2^ These therapies include drugs targeting Tumor Necrosis Factor (TNF)‐α, Interleukin (IL)‐17, and IL‐12/23. The newest class of biologics include drugs selectively targeting IL‐23 such as risankizumab and guselkumab which have been recently approved for the treatment of adults with moderate‐to‐severe plaque psoriasis who are candidates for systemic therapy.^3^, ^4^, ^5^ Guselkumab and risankizumab are human monoclonal‐antibodies that specifically inhibit intracellular and downstream signaling of IL‐23 by binding to its p19 subunit.^3^, ^6^, ^7^ Guselkumab was the first anti IL‐23 agent being available on the market for the treatment of moderate‐to‐severe psoriasis.^3^ Its efficacy and safety have been showed by VOYAGE‐1 and VOYAGE‐2, two phase‐III multicenter, randomized, double‐blind, placebo and comparator‐controlled clinical trials.^8^, ^9^ Moreover, recently real‐life studies confirmed trials results, showing guselkumab as a safe and effective treatment.^3^, ^10^, ^11^, ^12^, ^13^, ^14^, ^15^, ^16^ Risankizumab is another anti‐IL23 approved in both USA and Europe.^17^ Two phase‐III trials, UltIMMa‐1 and UltIMMa‐2, have reported a higher efficacy of risankizumab respect to ustekinumab^17^ whereas the phase‐III trial IMMvent showed its superiority against adalimumab.^18^ Both guselkumab and risankizumab efficacy and safety have been compared with anti‐TNF (adalimumab), anti‐IL‐12/23 (ustekinumab) and anti‐IL‐17 (secukinumab), showing promising results in PASI90 and PASI100 responses.^8^, ^9^, ^18^, ^19^ However, to date, study comparing guselkumab and risankizumab safety and efficacy in real world practice are still lacking. Real life studies are needed in order to verify the efficacy and safety of recently approved biologics for psoriasis in a more complicated setting of patients which are usually excluded from clinical trials. Herein, we performed a retrospective cohort study using real‐world data to indirectly compare the efficacy and safety of guselkumab and risankizumab in psoriasis patients.

## 2 METHODS

An Italian single‐center retrospective cohort study enrolling moderate‐to‐severe patients attending the Psoriasis Care Center of the University of Naples Federico II, Naples, Italy from 1^−^September‐2018 and 31‐December‐2020, was performed to indirectly compare guselkumab and risankizumab efficacy and safety. Inclusion criteria were: (i) moderate‐to‐severe plaque psoriasis diagnosed since at least 1 year; (ii) subjects starting guselkumab or risankizumab treatments and being treated for at least 12 weeks. Patients were treated with standard dose of guselkumab (100 mg sc administered by subcutaneous injection at Week 0 and Week 4, followed by a maintenance dose every 8 weeks) or with standard dose of risankizumab (two injections of 75 mg subcutaneously at Week 0, Week 4, and then every 12 weeks). At baseline, the following items were registered for each patient: (i) personal and demographic data; (ii) duration of psoriasis and eventual psoriatic arthritis (PsA); (iii) comorbidities; (iv) previous psoriasis systemic treatments; (v) Psoriasis severity using Psoriasis Area and Severity Index (PASI), Body Surface Area (BSA), and Dermatology Life Quality Index (DLQI) scores. At every follow‐up the following items were evaluated: psoriasis severity (PASI, BSA and DLQI), routine blood tests [blood count with formula, transaminases, creatinine, azotaemia, glycaemia, erythrocyte sedimentation rate, C reactive protein, total cholesterol and triglycerides levels protein electrophoresis], and adverse events (AEs). Safety was assessed by treatment‐emergent AEs, physical examinations and laboratory monitoring. Effectiveness data were analyzed using a last observation carried forward method, where if a patient dropped out of the study the last available value was ‘carried forward’ until the end of the treatment. The present study was performed respecting the Declaration of Helsinki.

### 2.1 Statistical analysis

Continuous variables were displayed as mean ± SD, whereas categorical variables or as number and proportion of patients. Demographic and clinical characteristics of the sample were described through absolute and relative frequencies (%), means and/or SDs where appropriate. *T*‐test and Chi‐squared test were used to compare the quantitative and qualitative characteristics of the populations treated with the two different drugs. A *p* value of <0.05 was considered statistically significant. All statistical analyses were performed using GraphPad‐Prism 4.0 (GraphPad Software Inc., La Jolla, CA, USA).

## 3 RESULTS

**FIGURE 1:**
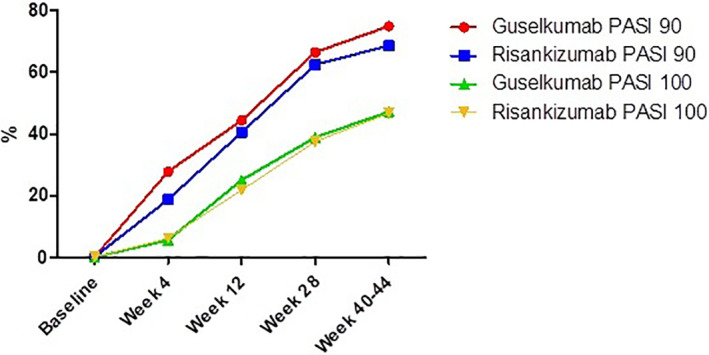
Comparison of PASI 90 and PASI 100 responses among guselkumab and risankizumab groups from baseline up to week 40 and 44 for risankizumab and guselkumab, respectively

A total of 68 patients were included in the study: 36 (52.9%) received guselkumab, while 32 (47.1%) patients received risankizumab. Guselkumab group comprised 21 males (58.3%) and 15 females (41.7%) with a mean age of 48.7 ± 17.9 years while risankizumab group was composed of 20 males (62.5%) and 12 females (37.5%) with a mean age of 44.8 ± 14.7 years (Table 1). Guselkumab and risankizumab groups were comparable for age, sex, psoriasis severity, comorbidities and previous systemic treatment except for mean psoriasis duration (*p* < 0.01) which was higher for guselkumab group (Table 1). Particularly, among comorbidities hypertension (38.9% vs. 46.8%,) was the most common one followed by dyslipidaemia (30.6% vs. 37.5%), diabetes (8.4% vs. 15.6%), and cardiopathy (16.7% vs. 9.4%); no significant differences were observed between the two groups (Table 1). Every single patient had received at least one conventional systemic treatment without any significant difference between groups (Table 1). Previous biologic treatment failure was reported in more than half of both groups (64% vs. 68%) without significant difference among them (Table 1). Mean PASI and BSA significantly reduced at each follow up for both guselkumab and risankizumab without significant statistical difference. Particularly, in guselkumab group, mean PASI score reduced from 16.1 ± 6.4 at baseline to 1.7 ± 0.9 at Week 28 (*p* < 0.001) up to 0.7 ± 0.8 at Week 44 (*p* < 0.001) (Figure 1). As regards risankizumab group mean PASI decreased from 13.5 ± 4.9 at baseline to 1.9 ± 0.8 (*p* < 0.001) at Week 28 (*p* < 0.001) up to 0.9 ± 0.4 at Week 40 (*p* < 0.001) (Figure 1). BSA showed an analogue trend (Table 1). Reported blood tests alterations were not relevant and did not significantly differed among the two groups. They were registered in 13.8% of guselkumab subjects [2 cases of mild transient hyperglycaemias; 1 case of hypertriglyceridemia; 2 patients showed increase of ESR; 1 case of liver enzyme elevation GOT: 419 n.v. 0–37 U/L GPT: 321 U/L n.v. 0–45 U/L and γ‐GT: 58 n.v. 10–39 U/L)] and in 15.6% of risankizumab patients [2 patients with a transient ESR of 19 and 20 mm/h (n.v. 0–12 mm/h); 1 patient hyperglycaemia; 2 patients hypertriglyceridemia]. In addition, potential registered AEs were similar among the groups: registered AEs were pharyngitis (8.4%), flu‐like illness (11.1%), and headache (5.5%) for guselkumab without requiring its discontinuation. In risankizumab group they were represented by upper respiratory tract infections (9.4%), headache (3.1%) and diarrhea (3.1%). Discontinuation rates were comparable between guselkumab and risankizumab. Three (8.4%) patients discontinued guselkumab, one patient due to liver enzymes elevation while the 2 remaining patients for PsA worsening. Particularly, the subject with liver enzymes alteration was already affected by chronic hepatitis C and 3 weeks after guselkumab discontinuation, liver enzymes returned to lower values (AST: 160, ALT: 117) while the two subjects with PsA worsening had already been failed for the same reason different anti‐TNFs and one anti‐IL17s. Two (6.2%) patients discontinued risankizumab due to secondary inefficacy (loss of PASI75 response after at least 12 weeks). No cases of serious AEs, injection site reaction, candida, malignancy, cardiovascular events were reported in both groups.

**TABLE 1:** Clinical data of patients treated with guselkumab and risankizumab

Treatment groups	Guselkumab	Risankizumab	*p*
Number of patients	36	32	
Sex			
Male	21 (58.3%)	20 (62.5%)	ns
Female	15 (41.7%)	12 (37.5%)	ns
Mean age (years)	48.7 ± 17.9 years	44.8 ± 14.7	ns
Mean duration of psoriasis	25.6 ± 10.9	16.6 ± 8.7	<0.01
Psoriatic arthritis	25% (*n* = 18)	37.5% (*n* = 12)	ns
Comorbidities			
Hypertension	38.9% (*n* = 14)	46.8% (*n* = 15)	ns
Dyslipidaemia	30.6% (*n* = 11)	37.5% (*n* = 12)	ns
Diabetes	8.4% (*n* = 3)	15.6% (*n* = 5)	ns
Cardiopathy	16.7% (*n* = 6)	9.4% (*n* = 3)	ns
Cardiac arrhythmia	0% (*n* = 0)	3.1% (*n* = 1)	ns
Depression	25.0% (*n* = 9)	21.9% (*n* = 7)	ns
Chronic hepatitis B infection	0% (*n* = 0)	3.1% (*n* = 1)	ns
Chronic hepatitis C infection	2.7% (*n* = 1)	0% (*n* = 0)	ns
GERD	0% (*n* = 0)	3.1% (*n* = 1)	ns
Hidradenitis suppurativa	0% (*n* = 0)	3.1% (*n* = 1)	ns
Previous conventional systemic treatments			
Cyclosporine	50.0% (*n* = 18)	56.2% (*n* = 18)	ns
Acitretin	30.6% (*n* = 11)	50.0% (*n* = 16)	ns
Methotrexate	44.4% (*n* = 16)	37.5% (*n* = 12)	ns
Nb‐UVB phototherapy	16.7% (*n* = 6)	9.4% (*n* = 3)	ns
Number of biologics previously failed			
*n* = 0 (bionaive)	36% (*n* = 13)	32% (*n* = 10)	ns
Bioexperienced	64% (*n* = 23)	68% (*n* = 22)	ns
*n* = 1	13.9% (*n* = 5)	18.7% (*n* = 6)	ns
*n* = 2	27.7% (*n* = 10)	25% (*n* = 8)	ns
*n* ≥ 3	22.2% (*n* = 8)	25% (*n* = 8)	ns
Adverse events			
Pharyngitis	8.4% (*n* = 3)	9.4% (*n* = 3)	ns
Flu‐like illness	11.1% (*n* = 4)	3.1% (*n* = 1)	ns
Headache	5.5% (*n* = 2)	6.2% (*n* = 2)	ns
Diarrhea	0% (*n* = 0)	3.1% (*n* = 1)	ns
Discontinuation rate	8.4% (*n* = 3)	6.2% (*n* = 2)	ns
Baseline			
Mean PASI	16.1 ± 6.4	13.5 ± 4.9	ns
Mean BSA	37.8 ± 14.4	28.4 ± 13.5	ns
Week 4			
Mean PASI	7.1 ± 3.9	5.9 ± 3.6	ns
Mean BSA	16.7 ± 8.9	12.3 ± 6.9	ns
PASI90	27.8% (*n* = 10)	18.7% (*n* = 6)	ns
PASI100	5.5% (*n* = 2)	6.2% (*n* = 2)	ns
Week 28			
Mean PASI	1.7 ± 0.9	1.9 ± 0.8	ns
Mean BSA	5.5 ± 2.9	6.2 ± 1.6	ns
PASI90	66.6% (*n* = 24)	62.5% (*n* = 20)	ns
PASI100	38.9% (*n* = 14)	37.5% (*n* = 12)	ns
Week 40–44^a^			
Mean PASI	0.9 ± 0.8	0.9 ± 0.4	ns
Mean BSA	2.1 ± 1.3	3.1 ± 1.0	ns
PASI90	75.0% (*n* = 27)	68.7% (*n* = 22)	ns
PASI100	47.2% (*n* = 17)	46.8% (*n* = 15)	ns

Abbreviations: BSA, body surface area; GERD, gastroesophageal reflux disease; PASI, psoriasis area severity index.

^a^ Week 40 and 44 for risankizumab and guselkumab, respectively.

## 4 DISCUSSION

Recent major research advantages revealed IL‐23/Th17 axis as the key immune pathway in psoriasis pathogenesis with IL‐23 playing a central role.^20^ Particularly, IL‐23 induces the production of proinflammatory mediators such as IL‐17A, IL‐17F, and IL‐22 by Th17 cells, leading to the activation and hyperproliferation of keratinocytes. These events result in amplification of the immune response, leading to the clinical features of the disease.^21^, ^22^ The efficacy and safety of guselkumab and risankizumab, two selective IL‐23 inhibitors, have been showed by several phase‐III trials which reported promising results in terms of PASI90 and PASI100 responses if compared with other biologics.^8^, ^9^, ^17^, ^18^, ^19^, ^23^, ^24^ Particularly, VOYAGE‐1 and VOYAGE‐2 trials reported PASI90 and PASI100 responses in 76.3% and 47.4%, respectively, at Week 16 and in 76.3% and 47.4% at Week 48, showing superiority compared to adalimumab.^8^, ^9^ In addition, NAVIGATE trial showed that patients unresponsive to ustekinumab derived significant benefit from switching to guselkumab while ECLIPSE trial demonstrated guselkumab superiority in terms of long‐term efficacy (PASI90 at Week 48) compared with secukinumab.^25^, ^26^ As regards risankizumab, UltIMMa‐1 and UltIMMa‐2 trials showed a higher efficacy compared to ustekinumab.^19^ Particularly, PASI90 was achieved by 75.3% of patients receiving risankizumab versus 42.0% receiving ustekinumab.^18^ Moreover, IMMvent study demonstrated risankizumab superiority respect to adalimumab, reporting that PASI90 response was achieved by 72% and 47% with risankizumab and adalimumab, respectively, at Week 16.^19^ In addition, IMMerge study reported risankizumab greater efficacy respect to secukinumab (PASI 90 at Week 52, 86.6% vs. 57.1%).^24^ Hence both guselkumab and risankizumab appear as two optimal treatments for psoriasis. However, due to their recent introduction on the market, data about their efficacy and safety in real world practice are needed in order to confirm the promising trials results. Indeed, real practice deal with more complicated patients (multiple comorbidities, polypharmacy, common previous biologic failure, etc) which usually do not meet trials inclusion criteria.^3^ Hence, we performed a retrospective real world practice indirect comparison of risankizumab and guselkumab efficacy and safety to highlight eventual differences or peculiarities. In our real‐world study mean PASI and BSA trends, as well as PASI90 and PASI100 rate responses resulted comparable among the two groups with no statistically significant differences being found at each follow‐up up to 40–44 weeks (Figure 1). These data are particularly significant for risankizumab since real world study are very scant. Indeed, several real life studies are already available for guselkumab: a 16‐week‐retrospective study on 180 psoriasis patients showed that no one has discontinued the treatment for inefficacy, and overall, 38.3% of patients achieved PASI‐100 and 50.6% PASI‐90.^13^ A Belgian 16‐week retrospective study reported PASI‐100, PASI‐90, and PASI‐75 responses in 32.1%, 55.4%, and 82.1% respectively.^14^ Moreover, a recent Italian 1‐year retrospective study reported PASI 75, 90, and 100 responses in 84.2%, 78.9%, and 63.2%, respectively at 12 months.^12^ On the other hand, to date fewer data about risankizumab real‐life efficacy have been reported due to its more recent approval and availability.^27^, ^28^ In an Italian single center 16 weeks, retrospective study, efficacy and safety of risankizumab resulted comparable to trials results. Particularly, PASI‐100 and PASI‐90 were achieved by 49.1% and 63.2% respectively.^27^ Moreover, in a previous single‐centre, prospective study we assessed risankizumab efficacy and safety in patients who had previously failed anti‐IL17, anti‐IL12/23 or anti‐IL23 inhibitor showing risankizumab as a promising therapeutic option in patients who failed these drugs.^17^ In addition, in a recent study risankizumab efficacy had been evaluated in patients who also initially failed guselkumab.^29^ Interestingly, the authors reported a mean PASI improvement of 90% at week 16, both in patients which previously failed guselkumab and in patients naïve to anti‐IL‐23 inhibitors.^29^ Another study comparing ixekizumab to IL‐23 inhibitors showed that PASI 75 response risk difference significantly favored ixekizumab over risankizumab at week 12 (*p* < 0.05), as did PASI90 response risk differences at week‐4 (*p* < 0.001), 8 (*p* < 0.001), and 12 (*p* < 0.05).^30^ Interestingly, our results showed that both guselkumab and risankizumab were effective in patients who previously failed other biologics (>50% of study population had previously failed at least one biologic drug). Previous biologic treatments, including anti‐TNF, anti‐IL17 and/or anti‐IL12/23, did not influence clinical outcomes in both guselkumab and risankizumab groups. In addition, as regards safety, our study showed both guselkumab and risankizumab as safe treatment options, with most frequent reported AEs being represented by pharyngitis (8.4%), flu‐like illness (11.1%), and headache (5.5%) in the guselkumab group while upper respiratory tract infections (9.4%), headache (3.1%) and diarrhea (3.1%) prevailed in the risankizumab group. None of these AEs required treatment discontinuation. Conversely, therapy discontinuation was necessary in 8.4% of patients treated with guselkumab (due to one case of liver enzymes elevation and two of PsA worsening) and in 6.2% of risankizumab patients (due to loss of PASI75 response after 12 weeks). Our study did not highlight any significant differences among these two drugs, being also in line with previous safety real data being available singularly. Indeed, as regards guselkumab, AEs have been reported in a variable rate between 0% and 30.4%.^10^, ^11^, ^12^, ^13^, ^14^, ^15^ including arthromyalgia, asthenia, infections (upper respiratory infection, tooth infection, pharyngitis), headache, syncope, anxiety.^10^, ^11^, ^12^, ^13^, ^14^, ^15^, ^16^ Reported discontinuation rates ranged from 0% to 9.8%, with most frequent causes of discontinuations represented by loss of efficacy, outbreak of PsA arthromyalgia, injection site reaction, panic attack, eczema and heart palpitation and malignancy (chronic lymphoid leukemia and Hodgkin's lymphoma).^10^, ^11^, ^12^, ^13^, ^14^, ^15^, ^16^ Even if real world practice data about risankizumab safety are scant, few published real‐life studies showed its excellent safety profile with reported AEs varying from 0% to 1.8% with upper respiratory tract infection being the most common reported AEs.^16^, ^27^, ^29^ Moreover, we wanted to highlight that the last 10 months of our study period were affected by the onset of the COVID‐19 pandemic which completely revolutionized dermatological clinical practice. During this period, many concerns have been raised among both patients and physicians whether it was advisable or not to stop biologic treatments.^31^ In this context, IL‐23 inhibitors showed to be a safe therapeutic option during the ongoing pandemic. Indeed, a retrospective multicenter cohort study including 6501 patients with chronic plaque psoriasis under biologics (*n* = 1691 with anti‐IL‐23), did not show any adverse impact of biologics on COVID‐19 outcome.^32^ Hence, a prophylactic treatment discontinuation in order to prevent infection risk or possible negative COVID‐19 outcomes is not required.^32^ In conclusion, our study revealed that in real world practice, guselkumab and risankizumab showed an elevated efficacy and safety profile, being comparable in terms of PASI90 and PASI100 responses as well as in AEs and discontinuation rates. However, more data are needed to confirm our results, with a larger study population for both groups, in order to evaluate the real impact and the exact role that both treatments may have in psoriasis management.

## 5 LIMITATIONS

The relatively small sample size and the retrospective nature of the survey may limit the generalizability of our results.

## CONFLICT OF INTEREST

G. Fabbrocini acted as a speaker or consultant for Abbvie, Amgen, Eli Lilly, Janssen, Leo‐Pharma, Almyrall, Novartis, and UCB. M. Megna acted as a speaker or consultant for Abbvie, Eli Lilly, Janssen, Leo‐Pharma, and Novartis. None of the contributing authors have any conflict of interest, including specific financial interests of relationships and affiliation relevant to the subject matter or discussed materials in the manuscript.

## AUTHOR CONTRIBUTIONS

All authors contributed equally in producing this work.

## Data Availability

Data sharing is not applicable to this article as no new data were created or analyzed in this study.
